# Psychometric properties of the Career Adapt-Abilities Scale–Short Form: evidence from Chinese elite athletes

**DOI:** 10.3389/fpsyg.2023.1230537

**Published:** 2023-08-29

**Authors:** Jin-Chuan Hu, Ning Su, Yanmei Huang, Yu-Duo Zou, Hao Liu, Jing-Dong Liu

**Affiliations:** ^1^Department of Physical Education, Sun Yat-sen University, Guangzhou, China; ^2^Physical Education School, Shenzhen University, Shenzhen, China; ^3^Department of Physical Education, Tianjin University of Science and Technology, Tianjin, China

**Keywords:** career adaptability, elite athletes, validity, reliability, factor structure

## Abstract

**Objectives:**

The present study aimed to examine the psychometric properties of the Chinese version of the Career Adapt-Abilities Scale-Short Form (CAAS-SF) among a sample of Chinese elite athletes.

**Methods:**

A sample of Chinese elite athletes (*n* = 770) was invited to participate in this study. First, the factor structure of the Chinese version of the CAAS-SF was examined, and six measurement models (CFA, H-CFA, B-CFA, ESEM, H-ESEM, and B-ESEM) were constructed and compared. Second, the internal consistency reliability of the Chinese version of the CAAS-SF was examined. Finally, structural equation modeling (SEM) was employed to assess the nomological validity of the Chinese version of the CAAS-SF.

**Results:**

The results showed that the hierarchical ESEM (H-ESEM) model best represented the factor structure of the CAAS-SF among Chinese elite athletes. It suggests that the higher-order factor of career adaptability explains the four distinctive but interrelated specific factors of concern, control, curiosity, and confidence. Cronbach's alpha coefficients (0.84–0.90), composite reliability (0.81–0.96), and coefficient omega hierarchical (0.855–0.94) of the Chinese version of the CAAS-SF were larger than the cutoff values, which suggest satisfactory reliability. The results of the SEM revealed that the higher-order factor of career adaptability was positively associated with career decision self-efficacy (β = 0.676, *p* < 0.001). This result is consistent with previous findings (r = 0.65, *p* < 0.01) and provided support for the nomological validity of the CAAS-SF among Chinese elite athletes.

**Conclusion:**

The findings of the present study indicated that the Chinese version of the CAAS-SF displayed satisfactory reliability and validity and could be used to assess the career adaptability of Chinese elite athletes. In addition, the total score of the CAAS-SF is suggested to be used in future research and practical works.

## Introduction

Career paths of people in various domains have been dramatically influenced by globalization, digitalization, rapid technological advancement, and economic turbulence over the past three decades. Increasing uncertainty and job insecurity in the labor market posits huge challenges for people, who are facing transitions between jobs, organizations, and occupations more frequently than ever before (Rudolph et al., 2017). Whether individuals can adapt fully to uncertain, ever-changing, and unpredictable working environments or not becomes a salient question throughout the process of individuals' career development. According to the career construction theory (CCT; Savickas, 2005), human development is driven by adaptation to the social environment with the goal of person–environment integration (Savickas and Porfeli, 2012). The career construction model of adaptation is the core of the CCT and explains the process of career construction by depicting a sequential process that people's adaptivity (willingness and readiness to adapt) positively influences their career adaptability (psychosocial resources), which, in turn, positively influences adapting responses (career behaviors), and further adaption results (career outcomes) (Savickas and Porfeli, 2012). Career adaptability is considered a central concept in CCT and plays a salient role in the adaptation process of career construction. Previous research in various domains has consistently demonstrated an important role of career adaptability by revealing its significant relationships with adaptivity, adapting responses, and adaptation results (Tian and Fan, 2014; Zacher, 2014, 2016; Guan et al., 2015; Hirschi and Valero, 2015; Hirschi et al., 2015; Rudolph et al., 2017; Pajic et al., 2018; Sverko and Babarovic, 2019; Hui et al., 2021). For example, it was found that individuals with higher career adaptability would experience a smoother career transition process (Brown et al., 2012), better satisfaction and wellbeing at work (Akkermans et al., 2018; Bollmann et al., 2019), lower intention to leave a job (Rasheed et al., 2020), and less stress at work (Soresi et al., 2012). Another study also revealed that career adaptability plays an important role in helping individuals to excel in his/her career (Rudolph et al., 2017).

Career adaptability has been defined as a psychosocial construct that denotes individuals' recourses for coping with current and anticipated tasks, transitions, and traumas in their occupational roles (Savickas and Porfeli, 2012). Therefore, career adaptability is a multidimensional construct including four components of concern, control, curiosity, and confidence, which are interrelated with but still distinctive from each other (Savickas, 2005; Savickas and Porfeli, 2012). Concern refers to the extent that people are interested in and are prepared for their future. Control refers to the extent that people are responsible for their future. Curiosity refers to the extent that individuals explore their alternative futures and the possible actions that may lead them to these futures. Confidence refers to the extent that individuals believe in themselves and their abilities to achieve their career goals (Savickas and Porfeli, 2012). Based on these definitions, Savickas et al. developed the Career Adapt-Ability Scale (CAAS; Savickas and Porfeli, 2012) and examined its psychometric properties among students and workers from 13 countries and regions. The CAAS includes 24 items measuring four dimensions of concern, control, curiosity, and confidence, with 6 items for each dimension. The factor structure of the CAAS was represented by a hierarchical model with career adaptability as a higher-order factor (global factor) and four dimensions as first-order factors (specific factors) (Savickas and Porfeli, 2012). The CAAS is the most widely used measure for assessing career adaptability and has been translated into various languages and validated among participants from different countries such as China (Hou et al., 2012), Switzerland (Johnston et al., 2013), Brazil (Teixeira et al., 2012), Lithuania (Urbanaviciute et al., 2014), Korea (Tak, 2012), and Croatia (Babarović and Šverko, 2016). Previous research has collectively demonstrated that the CAAS is a valid and reliable instrument. To facilitate research efficiency, especially in studies involving large surveys, Maggiori et al. (2017) developed and validated the Career Adapt-Abilities Scale-Short Form (CAAS-SF) by reducing the number of items of the CAAS. The CAAS-SF includes 12 items measuring four dimensions with 3 items for each. Previous research has provided support for its psychometric properties among samples in various countries (Australia, Perera and McIlveen, 2017; Turkey, Işik et al., 2018; China, Yu et al., 2019; Song et al., 2023; Switzerland, Urbanaviciute et al., 2019; Germany, Haenggli and Hirschi, 2020; and India, Pal and Jena, 2021). The CAAS-SF displayed the same hierarchical factor structure and similar psychometric qualities as the CAAS (Song et al., 2023).

The hierarchical factor structure of both the CAAS and CAAS-SF (Savickas and Porfeli, 2012; Maggiori et al., 2017) has been consistently replicated in previous research using the hierarchical confirmatory factor analysis (H-CFA) approach. CFA relies on a highly restrictive independent cluster model (ICM), in which relationships between items and non-targeted factors (cross-loadings) are assumed to be zero. However, the ICM-CFA assumptions might be unrealistic, especially for complex multidimensional instruments (Morin et al., 2016). Exploratory structural equational modeling (ESEM; Asparouhov and Muthén, 2009) was developed by incorporating the best elements of both CFAs and EFAs into the traditional SEM framework, which could be used to solve the abovementioned limitations of CFA. ESEM allows the presence of cross-loadings between items and their non-target factors through various matrix rotation methods such as geomin rotation and target rotation (Marsh et al., 2014). ESEM has been widely used to examine the factor structure of multidimensional psychometric constructs in previous research (Morin et al., 2016; Bhavsar et al., 2019; Xiao et al., 2019; Alamer, 2021). Moreover, ICM-CFA assumes distinct facets (specific factors; S-factors) without considering the existence of a potential unobserved “common core” (e.g., global factor; G-factor) that may be reflected by all items. To solve this limitation, bifactor CFA was proposed because it tests directly whether the G-factor co-exists with multiple S-factors (Chen et al., 2006; Reise et al., 2010). With the development of the ESEM, bifactor ESEM (B-ESEM) and hierarchical ESEM (H-ESEM) were proposed to fully capture the hierarchical and multidimensional nature of instruments (Morin et al., 2016). The estimation of B-ESEM models is feasible using target rotation or bi-geomin rotation (Reise, 2012; Morin et al., 2016). The estimation of H-ESEM models could be achieved using the ESEM-within-CFA approach, in which a specific first-order ESEM solution is allowed to be re-expressed using CFA (Morin et al., 2013). Although previous research has examined the factor structure of the CAAS using the B-CFA approach and found that the B-CFA model outperformed the CFA and H-CFA models of the CAAS (Matijaš and Seršić, 2021), no research has examined the factor structure of the career adaptability measures (e.g., CAAS or CAAS-SF) using ESEM, B-ESEM, and H-ESEM yet.

The CAAS-SF has been widely used among various populations from different cultures (including China) because of its advantages such as easy administration and time-saving (Maggiori et al., 2017; Akkermans et al., 2018; Yu et al., 2019; Lu, 2020). However, no previous research has examined its psychometric properties, especially the factor structure among the elite athlete population. Together with the limitations abovementioned regarding the factor structure of the career adaptability measures, the purpose of the present study was to examine the psychometric properties of the CAAS-SF among a sample of elite athletes from China. Specifically, the factor structure of the CAAS-SF was examined by comparing six measurement models (A, CFA model; B, HCFA; C, B-CFA; D, ESEM model; E, H-ESEM model; and F, B-ESEM model). Internal consistency reliability was examined using Cronbach's alpha (α) and composite reliability (CR). For the bifactor models (C: B-CFA; F: B-ESEM), additional reliability estimations were calculated. Finally, the nomological validity of the CAAS-SF was examined by assessing its association with career decision self-efficacy using structural equation modeling (SEM).

## Methods

### Participants

A sample of 770 Chinese elite athletes was invited to participate in this study by answering a set of questionnaires. The data of two participants were found incomplete with more than 20% of information missing and the data of 53 participants were found with a simple responding pattern (responses to all items were exactly same), therefore, the data of 55 participants were considered as invalid. Excluding these invalid data, data from 715 participants (334 female participants and 381 male participants; age: M = 17.19, SD = 4.06) were identified as valid and used for data analysis. Participants were from 15 sports, namely, basketball, synchronized swimming, gymnastics, diving, martial arts, badminton, table tennis, trampolining, weightlifting, fencing, water polo, volleyball, tennis, swimming, and track and field. The average training years was 7.17 years (SD = 3.98).

### Procedure

The inclusion and exclusion criteria of participants in this study were that all athletes who were training and competing at provincial and national levels at the moment of data collection would be qualified for this study. A convenient sampling method was used in this study. Athletes who were training at provincial sports training centers were contacted and invited to participate in this study. Participants who returned their informed written consent forms were asked to answer a set of questionnaires in a quiet room in the absence of their coaches or via online survey. For the participants who were below 18 years old, informed written consent was obtained from their coaches who were asked to act *in loco parentis* before data collection. All participants were informed that the survey was voluntary and that they had the right to withdraw at any time from the study. They were also told that it was an anonymous survey and that all information they provided would be confidentially kept and no third parties including their coaches could be able to access their responses. Data were collected between May and December 2022. All participants took part in the study voluntarily.

### Measures

#### Career adaptability

A Chinese version of the Career Adapt-Abilities Scale-Short Form (CAAS-SF; Maggiori et al., 2017; CAAS-SF China; Yu et al., 2019) was used to assess participants' career adaptability. The CAAS-SF China includes 12 items measuring four subscales of concern, control, curiosity, and confidence, which 3 items for each subscale. An example item is “becoming aware of the educational and vocational choices that I must make”. Responses were measured on a 5-point Likert scale ranging from 1 (not strong) to 5 (strongest). Previous research has consistently revealed that the CAAS-SF displayed satisfactory validity and reliability (Cronbach's alpha coefficients ranged from 0.89 to 0.95) in Chinese populations (civil servants, Yu et al., 2019; employees, Yang et al., 2019; college student-athletes, Lu, 2020; university graduates, Sou et al., 2021). In this study, Cronbach's alpha coefficients ranged from 0.84 to 0.90 and composite reliability ranged from 0.808 to 0.963.

#### Career decision self-efficacy

Career decision self-efficacy was assessed using eight items from the Career and Educational Decision Self-Efficacy Inventory developed by Ho and Sum (2016). An example item is “I am able to choose a career that will fit my interests”. Li et al. (2019) used the eight items to assess the career decision self-efficacy of university students in Hong Kong and the United States (Ho and Sum, 2016; Li et al., 2019). Responses were measured on a 5-point Likert scale ranging from 1 (strongly disagree) to 5 (strongly agree). Satisfactory internal consistency reliability of the scale has been reported in previous research (Hong Kong sample: 0.84; US sample: 0.89; Li et al., 2019). In this study, Cronbach's alpha coefficient of this scale was 0.85.

### Data analysis

**Figure 1:**
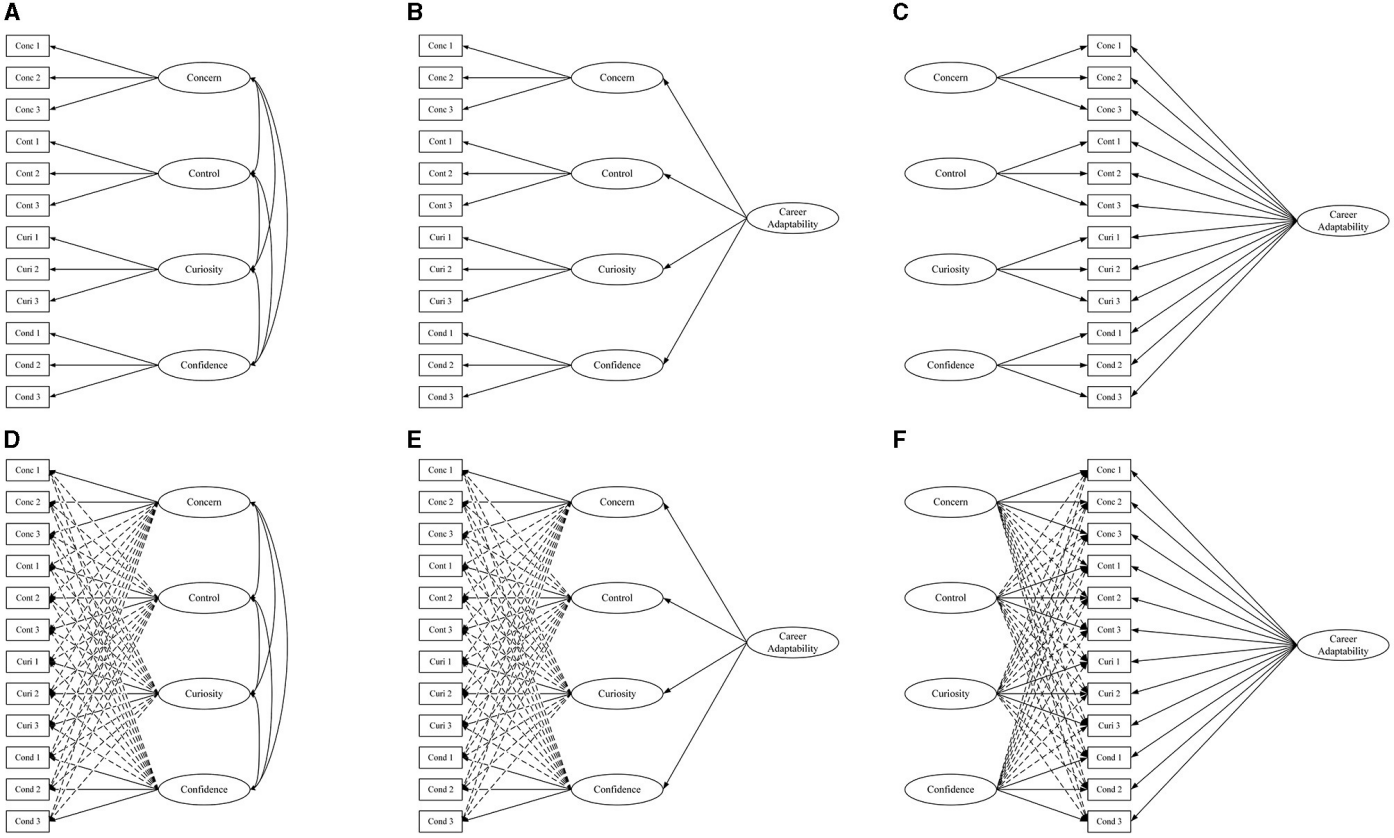
Measurement models of CFA **(A)**, H-CFA **(B)**, B-CFA **(C)**, ESEM **(D)**, H-ESEM **(E)**, and B-ESEM **(F)**.

SPSS (Version 23.0, Armonk, NY, United States: IBM Corp.) was used for data processing. For the model comparison, six measurement models (see Figure 1) were estimated using Mplus 8.0 (Muthén and Muthén, 1998–2014) based on the robust maximum likelihood (MLR) estimator. Specifically, in the CFA model (A), each item was allowed to load only on the factor it was assumed to measure and was not allowed to cross-load on other factors. This model includes four interrelated factors representing the subscales of the CAAS-SF described previously. In the H-CFA model (B), the four first-order factors were specified to be associated with a single higher-order CFA factor, and no residual correlations were specified between the four first-order factors. In the B-CFA model (C), all items were specified to load on both a global factor (G-factor) and their corresponding specific factors (S-factors), and the G-factor and the S-factors were specified to be not correlated with each other (Morin et al., 2015). In the ESEM model (D), the four factors were estimated as distinct but related first-order factors with all primary loadings and cross-loading estimated freely in an exploratory way (Morin et al., 2015). The H-ESEM model (E) was then estimated using the ESEM-within-CFA framework (Morin et al., 2013), in which all four first-order factors were specified to be correlated with a single higher-order factor with no residual correlations between the four first-order factors. Finally, the B-ESEM model (F) was estimated using bi-geomin rotation, in which a G-factor was defined by all items, and the four S-factors were defined by the same pattern of factor loadings (including primary loadings and cross-loadings) in the ESEM model.

Given the chi-square differences tests were sensitive to the sample size (e.g., Marsh et al., 2005), multiple common goodness-of-fit indices and information criteria were used to evaluate the fit of models including the comparative fit index (CFI), the Tucker–Lewis index (TLI), the root mean square error of approximation (RMSEA) with its 90% confidence intervals (CI), the standardized root mean square residual (SRMR), the Akaike information criteria (AIC; Akaike, 1987), the Bayesian information criteria (BIC; Schwartz, 1978), and the sample size-adjusted BIC (ABIC; Sclove, 1987). For the CFI and TLI, values >0.95 indicate a good model fit, but values of approximately 0.90 are acceptable. For RMSEA and SRMR, values < 0.08 or 0.06 indicate acceptable or good model fits, respectively (Hu and Bentler, 1999). The guidelines for nested model comparisons proposed by Chen et al. (2012) were followed in this study. When the sample size was larger than 300 (*n* = 715 in this study), a change in CFI (ΔCFI) of ≥ 0.005 accompanied by a change in RMSEA (ΔRMSEA) of ≥ 0.015 would suggest that the simpler model is better than the more complex model. Meanwhile, the suggestion by Marsh et al. (2010) was used that if the TLI or RMSEA is as good or better than the more complex model, the more parsimonious model would be preferred. For the information criteria (AIC, BIC, and ABIC), they in themselves are not helpful for model fit evaluation, but they are informative for model comparisons with a lower value reflecting a better fit to the data of one model in comparison with a model with higher values (Morin et al., 2016).

Cronbach's alpha coefficient (α) and composite reliability (CR) were used to evaluate the internal consistency reliability. Meanwhile, for the bifactor models, various statistical indices including coefficient omega (ω), omega hierarchical (ωh), and omega hierarchical subscales (ωhs), the percent of uncontaminated correlation (PUC) (available for the B-CFA model but not B-ESEM model), and explained common variance (ECV) were computed using the Bi-factor Indices Calculator (Dueber, 2017). Coefficient ω is a factor analytic model-based estimate of the reliability of unit-weighted test scores, which reflects the true score variance over observed score variances (Rodriguez et al., 2016a). Coefficient omega hierarchical (ωh) is the percent of total score variance attributable to a single general factor. The value of ωh >0.80 indicates that the total score could be considered unidimensional (Rodriguez et al., 2016b; Yilmaz Kogar and Kogar, 2022). The coefficient omega hierarchical subscale (ωhs) is the percent of subscale score variance attributable to a specific factor after removing the reliable variance due to the general factor. The value of ωhs < 0.50 indicates that most of the variance in the subscale score is due to the general factor and negligible unique variance is due to that specific factor (Reise et al., 2010). The PUC represents the percentage of covariance terms that only reflect variance from the general factor, and the ECV represents the proportion of all common variance explained by the general factor. According to Rodriguez et al. (2016b), when the ECV is >0.70 and PUC is >0.70, the common variance could be regarded as essentially unidimensional. Reise et al. (2013) argue that when the PUC value was lower than 0.80, the general ECV value >0.60, and Omega H larger than 0.70, it would indicate the presence of multidimensionality but was not severely enough to disqualify the interpretation of the instrument as unidimensional.

Finally, to be consistent with previous CAAS-SF studies, the nomological validity of the CAAS-SF was evaluated by examining its association (based on the optimal measurement model of CAAS-SF obtained in this study) with their theoretically relevant variable of career decision self-efficacy using SEM. Previous research has revealed low-to-moderate associations between the career adaptability subscale scores (rs = 0.34–0.44; Sou et al., 2022; Stead et al., 2022) and total score (rs = 0.65 to 0.66; Rudolph et al., 2017; Li et al., 2019) with the career decision-making self-efficacy. If similar results were observed in this study, the nomological validity of the CAAS-SF would be supported (Maggiori et al., 2017; Yu et al., 2019).

## Results

### Factor structure

The model fit indices and information criteria for the six models are shown in Table 1. All models demonstrated an acceptable fit to the data. Overall, ESEM models consistently outperformed their corresponding CFA models (e.g., ESEM vs. CFA, B-ESEM vs. B-CFA, and H-ESEM vs. H-CFA), which provided support for the ESEM solutions. Among ESEM models, the ESEM model and H-ESEM demonstrated similar model fit to the data (ΔCFI = −0.001, ΔTLI = −0.002, ΔRMSEA = 0.001, ΔSRMR = 0.002). According to Chen et al. (2005), compared with a first-order model with correlated factors, a second-order model would be preferred because it was more parsimonious and interpretable when an underlying construct was hypothesized to account for the common variance of first-order factors. Moreover, a closer inspection of the factor loadings revealed that, for the ESEM model, as shown in Table 2, the target loadings of nine items were larger than 0.40 (ranging from 0.501 to 0.813) with the non-target cross-loadings lower than 0.3 (ranging from 0.014 to 0.244). The target loading of 1 item was larger than 0.3 but smaller than 0.4 (Curiosity 3: observing different ways of doing things, 0.39) with the non-target cross-loadings smaller than 0.3 (ranging from 0.158 to 0.206). However, there were two items with non-target cross-loadings larger than their corresponding target loadings (Control 1: making decisions by myself; Confidence 1: taking care to do things well). Moderate inter-factor correlations (r_concern − control_ = 0.499, r_concern − curiosity_ = 0.397, r_concern − confidence_ = 0.421, r_control − curiosity_ = 0.576, r_control − confidence_ = 0.594, and r_curiosity − confidence_ = 0.661) were observed in the ESEM model. For the H-ESEM model, a similar pattern of factor loadings with the ESEM model was revealed as the H-ESEM was estimated using the unstandardized factor loadings derived from the ESEM model as starting values. However, the relationships between the four first-order specific factors and the second-order general factor in H-ESEM (λ_concernonCA_ = 0.551, λ_controlonCA_ = 0.764, λ_curiosityonCA_ = 0.757, λ_confidenceonCA_ = 0.813) were consistently higher than the inter-factor correlations among the four specific factors in ESEM. These results may imply that an underlying general factor may better explain the common variance of the four specific factors as the H-ESEM model did. It was found that although the B-ESEM model successfully converged and demonstrated a slightly better model fit than the H-ESEM model (ΔCFI = 0.007, ΔTLI = 0.012, ΔRMSEA = −0.014, ΔSRMR = −0.006), negative residual variance (Confidence 3: working up to my ability) was observed in the B-ESEM model. A closer inspection of the factor loadings of the B-ESEM model found that, as shown in Table 3, although all items significantly loaded on the G-factor of career adaptability (ranging from 0.639 to 0.811), only four items were found to significantly load on its corresponding target S-factor. These results imply that although the G-factor of career adaptability was well defined by most of the CAAS-SF items in the B-ESEM model, the four S-factors were poorly defined by their corresponding items. Collectively, these results suggest that the H-ESEM is more parsimonious and interpretable than the ESEM and B-ESEM models to represent the factor structure of the CAAS-SF measurement model, in which the higher-order factor of career adaptability explains the four distinctive but interrelated specific factors of concern, control, curiosity, and confidence.

**Table 1:** Goodness of fit statistics and information criteria of the CASS-SF measurement models.

**Model**	**χ^2^**	**p**	**df**	**CFI**	**TLI**	**RMSEA (90% CI)**	**SRMR**	**AIC**	**BIC**	**ABIC**
CFA	229.850	< 0.001	48	0.958	0.943	0.073 (0.063/0.082)	0.045	17,552.694	17,744.730	17,611.369
H-CFA	242.637	< 0.001	50	0.956	0.942	0.073 (0.064/0.083)	0.047	17,565.861	17,748.752	17,621.741
B-CFA	150.787	< 0.001	42	0.975	0.961	0.060 (0.050/0.071)	0.028	17,440.401	17,659.871	17,507.458
ESEM	61.301	< 0.001	24	0.991	0.977	0.047 (0.032/0.061)	0.011	17,341.215	17,642.985	17,433.418
H-ESEM	68.754	< 0.001	26	0.990	0.975	0.048 (0.034/0.062)	0.013	17,345.277	17,637.903	17,434.687
B-ESEM	29.429	0.0212	16	0.997	0.987	0.034 (0.013/0.053)	0.007	17,314.811	17,653.160	17,418.191

CFA, Confirmatory factor analysis; B, Bifactor model; H, Hierarchical model; ESEM, Exploratory structural equation modeling; χ^2^, Robust chi-square test of exact fit; CFI, Comparative fit index; RMSEA, Root mean square error of approximation; df, Degrees of freedom; SRMR, Standardized root mean square residual; CFI, Comparative fit index; TLI, Tucker-Lewis index; AIC, Akaike information criteria; BIC, Bayesian information criterion; ABIC, Sample size adjusted BIC.

**Table 2:** Standardized factor loadings of the CFA, H-CFA, ESEM and H-ESEM models.

**Factor**	**Items**	**Skewness**	**Kurtosis**	**CFA**	**H-CFA**	**ESEM**				**H-ESEM**			
				λ	λ	**F1(**λ**)**	**F2(**λ**)**	**F3(**λ**)**	**F4(**λ**)**	**F1(**λ**)**	**F2(**λ**)**	**F3(**λ**)**	**F4(**λ**)**
CONC	Conc 1	−0.123	−0.340	0.843	0.844	**0.737**	0.092	0.054	0.062	**0.741**	0.123	*0.037*	*0.047*
	Conc 2	−0.152	−0.398	0.878	0.879	**0.813**	0.07	0.06	0.064	**0.812**	0.07	0.061	0.064
	Conc 3	−0.274	−0.448	0.729	0.727	**0.39**	0.206	0.167	0.158	**0.399**	0.222	0.156	0.149
CONT	Cont 1	−0.148	−0.449	0.726	0.718	0.22	**0.568**	0.092	*0.014*	0.253	**0.583**	*0.08*	*−0.009*
	Cont 2	−0.361	−0.433	0.815	0.815	0.051	**0.697**	0.089	0.135	0.05	**0.695**	0.091	0.135
	Cont 3	−0.242	−0.543	0.842	0.847	*0.061*	**0.32**	0.512	*0.094*	0.073	**0.323**	0.481	0.119
CURI	Curi 1	−0.384	−0.534	0.881	0.881	0.117	*0.054*	**0.739**	*0.14*	0.116	0.053	**0.755**	0.139
	Curi 2	−0.284	−0.553	0.854	0.852	*0.058*	0.175	**0.501**	*0.244*	*0.059*	0.171	**0.474**	0.28
	Curi 3	−0.228	−0.358	0.753	0.753	0.108	0.202	**0.249**	0.333	0.112	0.2	**0.233**	0.351
COND	Cond 1	−0.399	−0.301	0.871	0.87	*0.045*	0.187	0.261	**0.509**	*0.044*	0.173	0.255	**0.531**
	Cond 2	−0.213	−0.473	0.812	0.814	0.146	0.302	*0.014*	**0.518**	0.157	0.288	*0.026*	**0.51**
	Cond 3	−0.240	−0.554	0.931	0.93	0.111	*0.058*	0.209	**0.705**	0.111	0.058	0.214	**0.701**

λ, Standardized factor loading; Target ESEM and Hierarchical ESEM factor loadings are indicated in bold; Non-significant parameters (*p* ≥ 0.05) are marked in italics; F1, Concern Factor; F2, Control Factor; F3, Curiosity Factor; F4, Confidence Factor.

**Table 3:** Standardized factor loadings of the B-CFA and B-ESEM models.

**Items**	**B-CFA**		**B-ESEM**				
	**FS(**λ**)**	**FG(**λ**)**	**FS1(**λ**)**	**FS2(**λ**)**	**FS3(**λ**)**	**FS4(**λ**)**	**FG(**λ**)**
Conc 1	0.585	0.613	**0.55**	*0.002*	*−0.018*	*−0.009*	0.639
Conc 2	0.639	0.646	**0.613**	*−0.006*	*−0.001*	*−0.011*	0.674
Conc 3	0.263	0.688	**0.213**	*−0.138*	*0.068*	*0.038*	0.696
Cont 1	*0.364*	0.675	*0.027*	***−0.267***	*−0.042*	*−0.063*	0.734
Cont 2	*0.368*	0.775	*−0.074*	***−0.087***	*−0.135*	*−0.082*	0.829
Cont 3	*0.075*	0.826	*−0.065*	***−0.046***	0.187	*−0.03*	0.822
Curi 1	0.075	0.863	*0.002*	*0.044*	**0.379**	*0.019*	0.847
Curi 2	0.768	0.822	*−0.009*	*0.277*	***0.084***	*−0.059*	0.82
Curi 3	0.053	0.745	*0.024*	*0.146*	***−0.025***	*0.027*	0.734
Cond 1	*0.117*	0.842	*−0.03*	0.174	*−0.027*	***0.103***	0.833
Cond 2	*0.122*	0.788	*0.034*	*0.031*	−0.123	***0.126***	0.797
Cond 3	*0.557*	0.887	*−0.012*	*−0.005*	*0.01*	***0.542***	0.881

λ, Standardized factor loading; Target Bifactor CFA and Bifactor ESEM factor loadings are indicated in bold; Non-significant parameters (*p* ≥ 0.05) are marked in italics; FS1, Concern Factor; FS2, Control Factor; FS3, Curiosity Factor; FS4, Confidence Factor; FG, Global Factor.

### Reliability

Cronbach's alpha coefficients of the four subscales ranged from 0.84 to 0.90, and the composite reliability (CR) values ranged from 0.808 to 0.963 (see Table 4). As mentioned earlier, although the B-ESEM model successfully converged and demonstrated good model fit, negative residual variance was observed. Additional reliability estimations were calculated for the B-CFA model, in which the reliability of the overall CAAS-SF (ω = 0.969), the G-factor of career adaptability (ωh = 0.921), and four S-factors (ωh concern = 0.868, ωh control = 0.855, ωh curiosity = 0.940, ωh confidence = 0.928) were found to be excellent. However, the ratio of the G-factor ωh and the overall ω (0.921/0.969 = 0.950) indicated that 95% of the reliable variance in the total score was attributed to the G-factor of career adaptability. Moreover, the omega hierarchical subscale coefficients for the concern, control, curiosity, and confidence subscales were found to be lower than 0.50 (ωhs_concern_ = 0.319, ωhs_control_ = 0.096, ωhs_curiosity_ = 0.113, ωhs_confidence_ = 0.084), which suggested that the percent of subscale score variance attributable to specific factors were small after removing the reliable variance due to the G-factor. The ECV value of the G-factor was 0.778, which suggested that the G-factor of career adaptability contributed 77.8% of the common variance, while the four specific factors contributed 22.2% of the common variance. This result suggested a fairly strong global factor that accounted for more than half of the common variance exceeding the cutoff value of 0.70. In other words, most part of the variance was explained by the G-factor, in which case the specific factors may have relatively low contribution. The PUC was 0.818 in this study, together with the EVC value of 0.778, indicating that the CAAS-SF was unidimensional. Collectively, these results suggest that the reliabilities of the global career adaptability and four subscales are satisfactory. However, it also a reminder that, in future research and practical works, the total score of the CAAS-SF rather than subscale scores should be used because most of the reliability variance was explained by the G-factor of the career adaptability.

**Table 4:** Descriptive statistics, Cronbach's alpha, composite reliability (based on H-ESEM) and additional indicators of B-CFA model.

**Subscales & total**	**M**	**SD**	**α**	**CR**	**B-CFA**		
					**ECV**	ω**h**	ω**hs**
G-Factor	-	-	-	0.963	0.778	0.921	-
CONC	3.382	1.011	0.849	0.873	0.393	0.868	0.319
CONT	3.582	0.974	0.840	0.808	0.136	0.855	0.096
CRUI	3.627	0.989	0.866	0.871	0.232	0.940	0.113
COND	3.630	0.972	0.901	0.940	0.138	0.928	0.084

M, Mean; SD, Standardized deviation; CONC, Concern; CONT, Control; CRUI, Curiosity; COND, Confidence; α, Cronbach's α; CR, Composite Reliability; ECV, Explained Common Variance; ωh, Omega hierarchical coefficients; ωhs, Omega hierarchical subscale.

### Nomological validity

**Figure 2:**
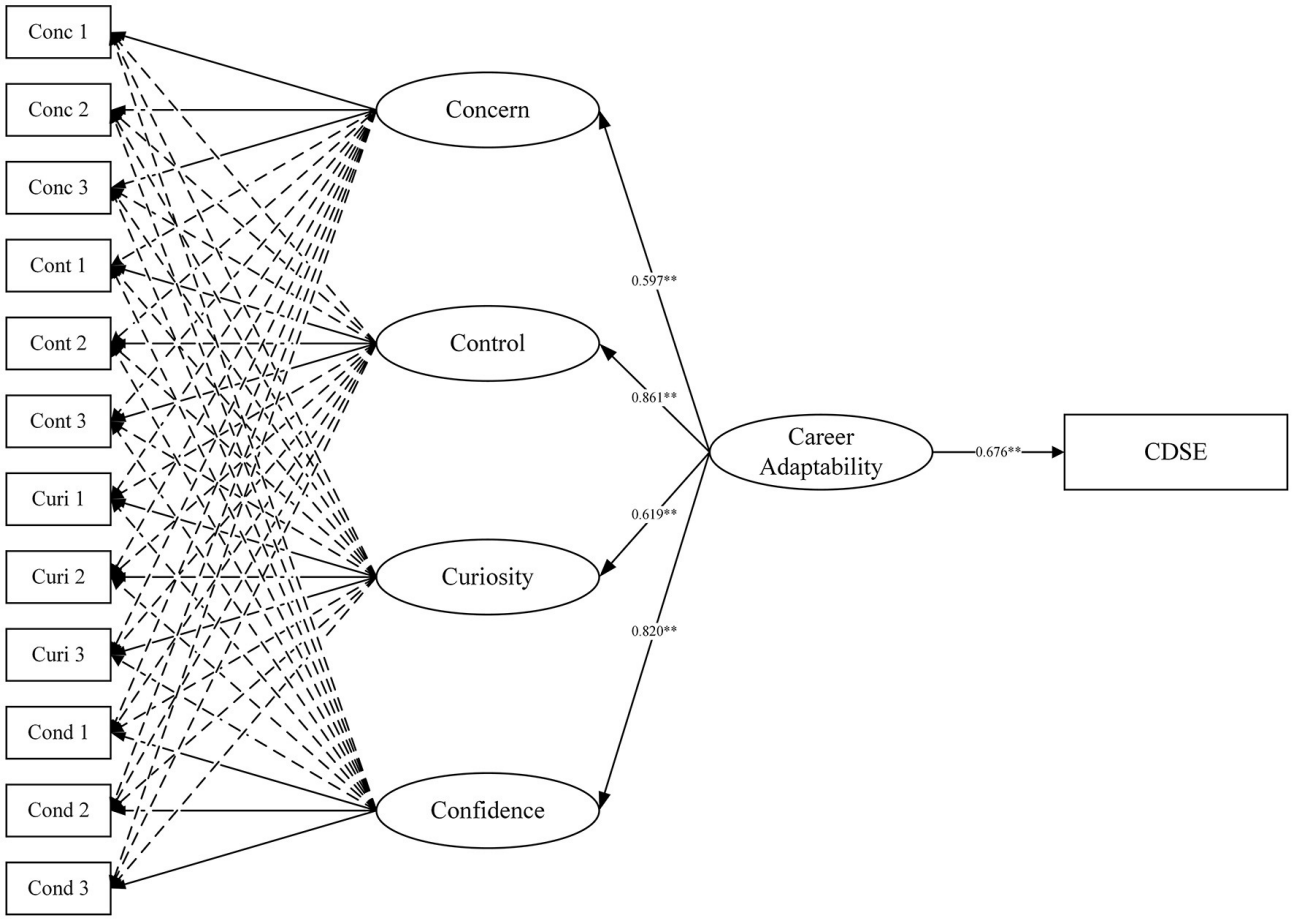
Relationship between career adaptability and career decision self-efficacy.

The results of reliability estimation in the B-CFA model suggested that the total score rather than the subscale score of the CAAS-SF would be more important and useful and latent or global variables should be used in structural equation modeling (SEM) analysis when exploring its associations with other variables. Therefore, SEM based on an H-ESEM solution was organized to examine the correlation between the CAAS-SF and career decision self-efficacy. Specifically, in this model, the higher-order factor of career adaptability was allowed to correlate with career self-efficacy (Figure 2). SEM results demonstrated an excellent model fit to the data (χ^2^ = 86.225, df = 38, *p* < 0.001, CFI = 0.990, TLI = 0.979, RMSEA = 0.042 (90% CI: 0.030–0.054), SRMR = 0.014). The higher-order factor of career adaptability was positively associated with career decision self-efficacy (β = 0.676, *p* < 0.001). The model explains a 45.7% variance in career decision self-efficacy. This result was consistent with previous results (r = 0.65, *p* < 0.01) and provided support for the nomological validity of the CAAS-SF among elite athletes from China.

## Discussion

Over the past 30 years, career adaptability has received extensive attention in the fields of career development and counseling. The development of the career adaptability measures (e.g., CAAS and CAAS-SF) has boosted a large number of studies on the topic in various settings and cultures to explore its relationships with predictors and consequences as well as its mediating role between variables. Previous research has demonstrated that career adaptability plays a salient role in the career development process of individuals from various fields. However, the evidence from elite athlete populations on career adaptability is still scarce, especially elite athletes from China. Meanwhile, although previous research has provided support for the psychometric properties of career adaptability measures (e.g., CAAS and CAAS-SF), the factor structures of these measures deserve further exploration because previous research has mainly relied on the ICM-CFA approach, which has been criticized as too restrictive and unrealistic in the real world. Specifically, CFA does not allow items to cross-load on non-target factors, which is not the case for most multidimensional measures including the CAAS-SF. In addition, previous research has specified the factor structure of the CAAS and CAAS-SF as the hierarchical model (second-order model); however, researchers have employed both the subscale scores and total scores of career adaptability measures to explore its relationships with other variables without supporting evidence regarding whether the subscale scores or total score should be used. These limitations may hamper and confound future research on the topic and application of career adaptability in practice. Therefore, to tackle the abovementioned limitations, the purpose of the study was to examine the psychometric properties of the CAAS-SF among a sample of elite athletes from China, with a special focus on its factor structure. A better understanding of the factor structure of the CAAS-SF in Chinese elite athletes has important implications for future research and practice on the topic of career adaptability in the Chinese elite athlete population.

This study contributes to the growing literature on career adaptability by examining the factor structure of CAAS-SF using ESEM among a sample of Chinese elite athletes. To the best of our knowledge, this is the first study to explore the factor structure of the CAAS-SF by comparing various CFA (CFA, B-CFA, and H-CFA) and ESEM (ESEM, B-ESEM, and H-ESEM) measurement models. In model comparisons, it was found that the B-CFA model outperformed the H-CFA and the first-order CFA. The results are consistent with previous findings on CAAS (Matijaš and Seršić, 2021). Although the B-CFA model allows testing whether the potential general factor may coexist with specific factors, the nature of the ICM-CFA not allowing items to cross-load on non-target factors may result in the biased estimation of the factor structure, especially on the inter-factor correlations. According to Marsh et al. (2009), ESEM is an alternative approach that could be used to address the abovementioned limitation of CFA. Our further ESEM-based analyses (first-order ESEM, B-ESEM, and H-ESEM) suggested that all ESEM models outperformed their corresponding CFA models (CFA, B-CFA, and H-CFA), respectively, which provided support for the application of ESEM in complex multidimensional psychometric constructs (Morin et al., 2016). Although the H-ESEM model and the ESEM model demonstrated satisfactory and similar model fit to the data, the H-ESEM model was more parsimonious. The B-ESEM model successfully converged and demonstrated a slightly better model fit than the H-ESEM model did, and negative residual variance was observed. Therefore, the H-ESEM model was preferred. Further inspection of factor loadings of the B-ESEM model revealed that all of the CAAS-SF items significantly loaded on the G-factor but only four items significantly loaded on their specific factors. The results in this study suggested that most of the variance of items were explained by the G-factor of career adaptability but not specific factors. In other words, most of the CAAS-SF items are good indicators of the G-factor of career adaptability but not valid indicators of specific factors. Factor loadings derived from ESEM and H-ESEM displayed a similar pattern because the H-ESEM was estimated using the unstandardized factor loadings derived from the ESEM model as starting values. Cross-loadings of two items were found to be larger than their target loadings, which reminds us that the two items may not accurately capture the nature of their corresponding specific factors. More research is needed to further explore the function of the two items among Chinese elite athletes, and modifications or revisions on the two items may be necessary. Furthermore, factor loadings of the four first-order factors to the higher-order factor in the H-ESEM model were moderate to high (0.551–0.813), which imply that common variance among the four factors could be better explained by the underlying higher-order factor. Collectively, our results suggest that the H-ESEM model is a more appropriate representation of the factor structure of the CAAS-SF among elite athletes from China because the four distinctive but interrelated factors could be explained by the underlying factor of career adaptability. These results advanced our understanding of the factor structure of the CAAS-SF by providing support for the hierarchical nature of the CAAS-SF.

Reliabilities of the four subscales were found satisfactory with all Cronbach's alpha values above 0.80 (0.84–0.90) in this study. Meanwhile, the composite reliability (CR) values based on the H-ESEM model also indicate good reliability for both the overall CAAS-SF scale and the four subscales, with all CR values exceeding 0.80 (ranging from 0.808 to 0.940). These results are consistent with the findings of previous studies (Garcia et al., 2019; Spurk et al., 2019; Yu et al., 2019). Although the B-ESEM model converged, negative residual variance was observed, which suggests that there are some problems with the model. Therefore, additional indicators of the B-CFA model (ω, ωh, ωhs, and ECV) were calculated, which provided informative evidence for the reliability estimation of the CASS-SF. Specifically, the reliabilities of the overall CAAS-SF (ω = 0.969) and the global factor of career adaptability (ωh = 0.921) were excellent. However, after removing the reliable variance due to the G-factor, the omega hierarchical subscale coefficients for the concern, control, curiosity, and confidence subscales were lower than 0.50 (ωhs = 0.084–0.319). Moreover, the values of ECV and PUC were higher than 0.70 (ECV = 0.778; PUC = 0.818), which reflected a unidimensional structure. Collectively, the results of Cronbach's alpha coefficients and composite reliability suggested that the CASS-SF displayed satisfactory reliability. However, additional estimation of reliability obtained in the B-CFA model reminded us that the total score of the CAAS-SF rather than subscale scores should be used in future research and practical works.

Previous research has revealed moderate associations between the career adaptability subscale scores (rs = 0.34–0.44; Sou et al., 2021; Stead et al., 2022) and the total score (r = 0.66; Li et al., 2019) with the career decision-making self-efficacy. Given the findings of the B-CFA model suggested that total score rather than subscale scores should be used, the nomological validity of the CAAS-SF was evaluated by examining the association of career adaptability total score with the career decision self-efficacy using SEM. A moderate relationship between the career adaptability total score and the career decision self-efficacy was observed in this study, which is consistent with previous findings (Li et al., 2019). Therefore, we concluded that the nomological validity of the CAAS-SF was supported.

## Limitations and recommendations

Although the present study provided preliminary psychometric evidence for the CAAS-SF in elite athletes from China and contributes to the literature on career adaptability research in sports, several limitations should be acknowledged. First, convenience sampling was employed in this study, which may limit the generalizability of the results. Future researchers are encouraged to recruit participants using a stratified sampling approach to include more athletes from various regions to better represent the population. Second, the factor structure of the CAAS-SF was initially examined by comparing six measurement models. Future studies are encouraged to further examine whether the factor structure would be invariant across groups, such as sex, sports levels, and age. Finally, test–retest reliability was not examined in this study. Future studies are encouraged to shed light on this issue.

## Conclusion

Collectively, our study provided initial support for the psychometric properties of the CAAS-SF in a sample of Chinese elite athletes. The H-ESEM model was found to be more appropriate to represent the factor structure of the CAAS-SF. The CAAS-SF demonstrated satisfactory validity and reliability and could be used to assess the career adaptability of athletes from China. More importantly, in practice, the total score of CAAS-SF should be used. Collectively, the validation of the CAAS-SF among Chinese elite athletes would help researchers and career counselors in competitive sports in the Chinese context to evaluate the career adaptability of Chinese elite athletes. For researchers, they can use the instrument to further explore factors that may contribute to the development of career adaptability as well as its consequences among Chinese elite athletes. For career counselors, the Chinese version of CAAS-SF would provide them with a valid tool to evaluate the changes in career adaptability of Chinese athletes, especially in their career counseling practices to enhance career adaptability using various intervention strategies.

## Data availability statement

The raw data supporting the conclusions of this article will be made available by the authors, without undue reservation.

## Ethics statement

The studies involving humans were approved by Institutional Review Board at the Sun Yat-sen University. The studies were conducted in accordance with the local legislation and institutional requirements. The participants provided their written informed consent to participate in this study.

## Author contributions

J-DL and NS: conceptualization, resources, and writing–original draft preparation. J-CH, NS, YH, and J-DL: methodology and formal analysis. HL and Y-DZ: validation. NS, Y-DZ, and HL: investigation. J-CH, NS, and HL: data curation. J-DL and HL: writing—reviewing and editing and supervision. All authors meet the criteria for authorship according to their contributions to the manuscript. All authors contributed to the article and approved the submitted version.
